# Spatial-Tunable Au Nanoparticle Functionalized Si Nanorods Arrays for Surface Enhanced Raman Spectroscopy

**DOI:** 10.3390/nano10071317

**Published:** 2020-07-04

**Authors:** Dongdong Lin, Kunjie Dai, Tianxiang Yu, Wenhui Zhao, Wenwu Xu

**Affiliations:** Department of Microelectronic Science and Engineering, School of Physical Science and Technology, Ningbo University, 818 Fenghua Road, Ningbo 315211, China; 176000229@nbu.edu.cn (K.D.); 176001983@nbu.edu.cn (T.Y.); zhaowenhui@nbu.edu.cn (W.Z.)

**Keywords:** detection, SERS, Au nanoparticles, Si nanorods, surface plasmon resonance

## Abstract

In this study, hexagonal-packed Si nanorods (SiNRs) arrays were fabricated and conjugated with Au nanoparticles (AuNPs) in different spatial distributions for surface-enhanced Raman spectroscopy (SERS). The AuNPs were functionalized on the bottom of SiNRs (B-SiNRs@AuNPs), top of SiNRs (T-SiNRs@AuNPs) and sides of SiNRs (S-SiNRs@AuNPs), respectively. Our results demonstrated that the SiNRs conjugated with AuNPs on the sides achieved high reproducibility in detection of R6G molecules, while the AuNPs on the top of the SiNRs obtained the strongest Raman enhancement. In addition, the substrate with S-SiNRs@AuNPs obtained the highest spatial uniformity of enhancement. The finite-difference time-domain simulation gave further evidence that the incident light could be confined in the space of SiNRs arrays and yield a zero-gap enhancement coupled with the AuNPs. Our study provided a spatially tunable SiNRs@AuNPs substrate with high sensitivity and reproducibility in molecular detection.

## 1. Introduction

Surface-enhanced Raman spectroscopy (SERS) has developed quickly since its discovery into a powerful molecular analytical tool [1,2,3]. The method has been widely used in various areas, such as analytical chemistry, medical science, and bio-systems [4,5,6]. The Raman scattering is dramatically enhanced by the electromagnetic (EM) field on some noble metal substrates. The areas with particularly large enhancement of EM (named hot spots) are found to be active positions for SERS detection [7,8]. As a result, if the target molecules are placed at the position of a hot spot, it will yield a comparably high Raman signal (several ordered levels) with the unique fingerprint of molecular structures. Clusters of noble metal nanoparticles, such as Au and Ag nanoparticles, have shown high SERS sensitivity with the sub-10 nm interstructure gaps [9,10]. Moreover, SERS detection is a label-free method that has great advantages compared with other labeling methods [11].

Extensive research has been carried out on SERS-active substrates for molecular analysis. One of the methods is the synthesis of metal nanostructures (nanoparticles, nanorods, etc.) through chemistry approaches. For example, assembly of Au nanoparticle (AuNPs) superstructures [12], topographical control of capillary assembly of Au nanorods [13], metal liquid-like Ag nanoparticles [14], and chitosan nanofibers conjugated with AuNPs [15]. However, the traditional SERS substrates (such as particle colloids) usually acquire randomly distributed hot spots, which results in heterogeneous enhancement. In addition, the application of SERS-based sensor detection requires the features of robustness, stability, high enhancement, uniformity and reproducibility. One solution could be the fabrication of regular periodic nanostructures for SERS, with methods including chemical etching and electron-beam/laser lithography. Chemical etching is one of the most popular and low-cost methodologies, and is widely used in SERS fabrication. For instance, self-assembly metal nanoparticles physically aggregate into large hexagonally close-packed arrays for high-sensitivity and high-fidelity SERS [16,17]. SiO_2_-isolated Ag islands on ordered arrays of polystyrene colloidal particle templates have been used as SERS substrate [18]. Wet etching-assisted PS colloidal lithography has been used to fabricate metal nanodisk and nanohole arrays [19,20]. In previous studies, highly dense and uniform arrays of Au-capped Si nanopillars on a 300 mm wafer level were fabricated by deep UV immersion lithography [21]. Au/Ag-coated periodic nanostructures on Si/SiO_2_ wafers have been achieved by low-cost and large-area laser-assisted nanoreplication [22]. Two-dimensional gratings have been fabricated by laser interference lithography on gold evaporated nanopillars [23]. In comparison, electron-beam/laser lithography could be more precise and editable with respect to fabrication, but the results would be limited in size and more expensive. Overall, those creatively fabricated SERS substrates incorporate a designed ordered narrow gap in the metal nano/microstructures and achieve high sensitivity and reproducibility. 

Other than the design of narrow gap between metal nanostructures, the manipulation of incident light by three-dimensional nanostructures has also been considered as a potential strategy [24]. For example, hexagonal-packed silver-coated silicon nanowire arrays could be made as a nanogap-free SERS system with long-range electric fields [25]. In contrast to the dipolar electric fields of metal nanoparticles by localized surface plasmon resonance (LSPR), which exhibited a monomer-to-monomer active spot, there are spatial enhancements in the electric fields on every silicon nanowire surface. The incident light could be coupled in the space of ordered nanowire and exhibit enhanced wide-range antinodes [26,27]. Whispering gallery mode (WGM) resonance can also enhance Raman scattering by increasing the reflection of electromagnetic waves in the cavity [28,29]. Repeated and multiple light scattering in photonic microarrays can enhance the matter–light interaction. In another case, the self-assembly of AuNPs on a 3D nanocup array structure generated strong LSPR effects due to additive optical coupling between two plasmons [30]. Those multiple coupling approaches were able to produce enhancements on the order of 10^7^~10^9^ over a wide area. However, the multiple coupling effects are associated with the material, the morphology of nanostructure, the location of the metal nanoparticles, and so on. As a result, it remains to be clearly investigated, and could offer further improvements in SERS performance.

In this paper, a low-cost substrate consisting of AuNPs and SiNRs was easily synthesized and fabricated in the absence of complicated lithography. To achieve the large-area ordered hexagonal-packed SiNRs substrate, sphere lithography and metal assisted chemical etching methods were used. The SiNRs substrate were further functionalized with homogeneous AuNPs. As a result of the periodic SiNR arrays and widely functionalized AuNPs, we achieved highly sensitive and reproducible SERS signals with high spatial uniformity at the centimeter scale. To investigate the multiple coupling effect between plasmonic particles and SiNRs arrays, The AuNPs were functionalized on the bottom of SiNRs (B-SiNRs@AuNPs), top of SiNRs (T-SiNRs@AuNPs) and the sides of SiNRs (S-SiNRs@AuNPs), respectively. The light could be trapped in different positions of the substrate by the designed ordered SiNRs arrays. In comparison, the S-SiNRs@AuNPs substrate obtained the highest reproducibility and spatial uniformity by creating a long decay length network. The relative standard deviation reached as low as 6.2% over 30 points across the substrate. The T-SiNRs@AuNPs substrate achieved the strongest SERS activity, due to the high density of the narrow AuNPs gap coupled with the localized incident light. Overall, we provided different AuNPs and SiNRs conjugation modes, which could be selectively used in SERS detection.

## 2. Materials and Methods 

### 2.1. Materials

The polystyrene spheres (PS, 500 nm in diameter) were purchased from Duke Scientific (Fremont, CA, USA) and the Si wafers were purchased from MTI (Sichuan, China). The chemicals for fabrication (Acetone, methanol, H_2_SO_4_, H_2_O_2_, HF) were purchased from Sinopharm Chemical Reagent (Shanghai, China). HAuCl_4_, trisodium citrate, R6G molecule were purchased from Sigma-Aldrich Corporation (Shanghai, China). All of the deionized water was obtained from the Millipore system.

### 2.2. Fabrication of SiNRs@AuNPs

AuNPs were synthesized using citrate reduction of HAuCl_4_. All of the glass vessels were cleaned with aquaregia for about 30 min and thoroughly rinsed with fresh deionized water before use. A 40 mL aqueous solution of 0.01% HAuCl_4_ was stirred mechanically in an oil bath at 130 °C. After boiling, 400 mL of 1% trisodium citrate solution was added, and the solution was stirred vigorously for the set time. The size of AuNPs was controlled through the incubation time. The size and monodispersity of the AuNPs were checked by scanning electron microscopy. In this work, AuNPs were synthesized with a diameter of 20 nm. For Ultraviolet-visible (UV-Vis) absorption measurements, an Agilent 8453 UV-Visible/NIR spectrophotometer with micro-quartz cuvettes with an optical path length of 10 mm was used. 

The methods of fabricating ordered SiNRs were reported in our previous work [31]. Firstly, a monolayer of polystyrene spheres (PS) with a diameter of 500 nm were self-assembled on a chemically cleaned planar Si wafer. Then the samples were baked for 3 min to remove the residual Deionized (DI) water and other adsorptions at 80 °C. Next, the PS covered samples were cut into small pieces and etched by reactive ion etching (RIE) with O_2_ gas (Samco, Japan). The gas flow rate was set at 30 sccm, with a power of 30 W, and a chamber pressure of 74 mTorr. The etching time was set at 280 s. Afterward, a 20 nm Au layer was sputtered onto the PS samples using an ion sputtering apparatus (Cressington, 108Manual, Watford, UK) at a constant current of 10 mA. After the deposition of Au, SiNRs were obtained by wet etching using HF and H_2_O_2_ with a volume ratio of 4:1. The etching solution was stirred, and the length of the nanorods could be adjusted by controlling the etching time. Finally, the PS were removed by RIE with O_2_. The remaining Au layer was removed by soaking the sample in KI/I_2_ mixed solution for 24 h (10 g of KI, 2.5 g of I_2_, and 100 mL of DI water). 

To decorate the AuNPs onto the surface of SiNRs, hybrid composites were prepared via the “grafting onto” strategy. Firstly, the fabricated SiNRs substrate was functionalized with a Si hydroxy group. The SiNRs substrate was immersed in 5 mL of ethanol which contain 50 μL of 3-Mercaptopropyl-trimethoxysilane (MPTS) and 10 μL of aqueous NH_4_OH (27%). The immersed SiNRs substrate was covered with aluminum-foil paper for 24 h at 25 °C, and then the MPTS-treated substrate was washed with ethanol and DI water several times. For the coating of AuNPs, the MPTS-treated SiNRs substrate was thoroughly immersed in 1 mL of an AuNPs solution (~1 nM) for 4 h at 37 °C. The resulting S-SiNRs@AuNPs was washed with water several times. Here, the B-SiNRs@AuNPs substrate could easily be obtained with a non-MPTS-treated SiNRs substrate. The T-SiNRs@AuNPs substrate should be immersed in AuNPs solution (~0.1 nM) with high temperature (50 °C) for quick desiccation. The higher temperature accelerates the evaporation of the AuNPs solution, leading to a dry film before the wetting of the AuNPs solution into inter SiNRs space. Finally, the substrates were exposed to UV light for 30 min and washed with water to remove the residual MPTS by photodecomposition. 

### 2.3. Characterization

The monodispersity of the AuNPs and SiNRs@AuNPs substrate was checked by scanning electron microscope. For UV–Vis absorption measurements, an Agilent 8453 spectrophotometer with cuvettes with an optical path length of 10 mm was used. 

The Raman spectra were obtained using a Jobin Yvon HR-Evolution 2 system with different excitation wavelength. The laser beam was focused on a spot with a diameter of 1~5 μm by the microscope objective. To obtain SERS spectra, 2 μL of R6G solution with different concentrations was dropped onto the different AuNP@SiNR substrate. In the spot-to-spot Raman measurements, a scanning matrix of 1000 μm with 10 points was set and repeated three times.

### 2.4. Finite-Difference Time-Domain Simulation

Lumerical FDTD Solution (version 8.12.590, Lumerical Inc., Vancouver, Canada) was used to perform FDTD simulations. the SERS substrate was placed in vacuum with the plane wave source on the top. Mesh enclosing of the model was set to 0.5 nm for AuNPs. The *z*-axis boundary of the structure region was set to perfect matching layer, while the *x*- and *y*-axes were set to periodic. Near-field electrical intensity profiles were obtained from a *z*-normal 2D monitor and *y*-normal 2D monitor. The optical data of Au is selected from Johnson and Christy [32]. 

## 3. Results

**Figure 1:**
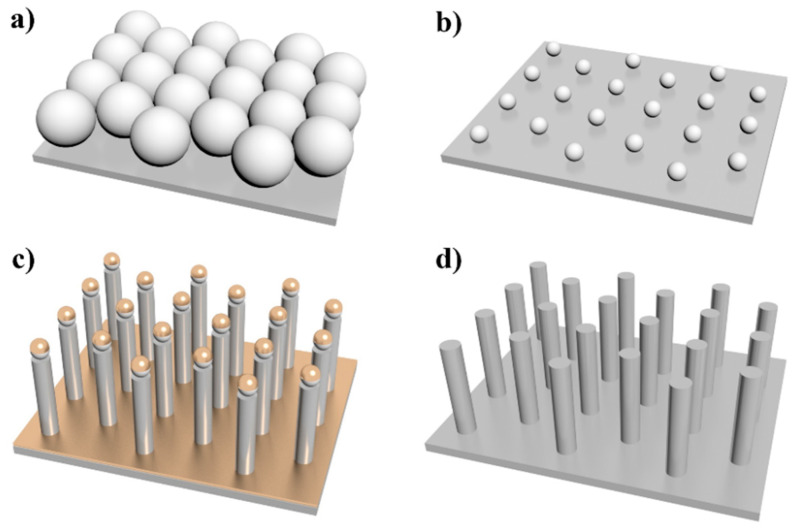
Schematic illustration of the SiNRs fabrication process. (**a**) Close-packed monolayer of PS nanospheres on Si wafer; (**b**) small PS array by reactive ion etching; (**c**) Au deposition and metal-assisted chemical etching into SiNRs; (**d**) remove Au.

Ordered hexagonally arranged SiNR arrays were fabricated by nanosphere lithography and metal-assisted chemical etching (Figure 1). The detailed procedure is introduced in the Materials and Methods section. Briefly, a large area of uniform polystyrene spheres (PS) monolayer (hexagonally packed) was prepared on the chemically clean Si wafer using the gas–liquid interface assembly strategy (Figure 1a). The scale of the PS-covered Si substrate could be as large as centimeter. Next, the diameter of the PS was reduced by reactive ion etching (RIE), as shown in Figure 1b. Then Au deposition and metal-assisted chemical etching were applied to create ordered SiNRs (Figure 1c). Finally, all of the Au and PS was removed by RIE and KI/I_2_ immersion (Figure 1d). The wet etching assisted colloidal lithography strategy could provide large-scale SiNRs substrates easily compared with electron beam lithography. The size of the hexagonal-packed SiNRs reached 2 × 3 cm^2^, with a diameter of ~200 nm and a length of ~0.8–1.3 μm in single nanorod. The next step is the conjugation with AuNPs (detailed steps are described in the Materials and Methods section), which is the key step in the substrate fabrication procedure. 3-mercaptopropyltrimethoxysilane (MPTS) was selected as a simple structure that can be easily removed by photolysis. Three −OCH_3_ are strongly connected with silicon hydroxyl by chemical reaction.

**Figure 2:**
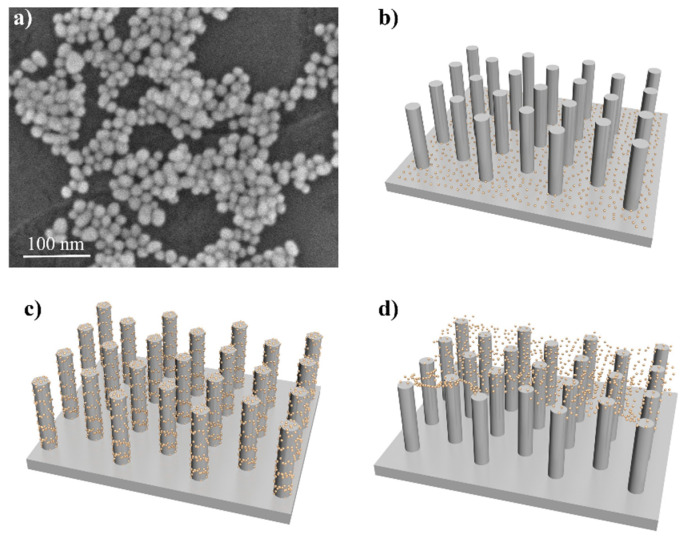
Conjugation of AuNPs on different sites of SiNRs. (**a**) SEM image of synthesized AuNPs; (**b**) B-SiNRs@AuNPs substrate; (**c**) S-SiNRs@AuNPs substrate; (**d**) T-SiNRs@AuNPs substrate.

**Figure 3:**
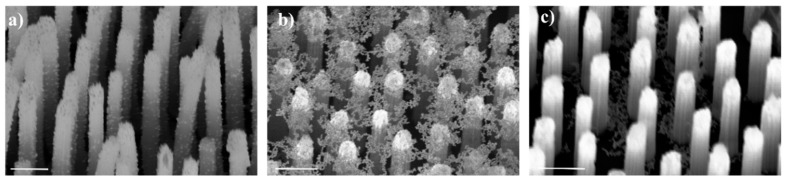
SEM images of three types of SERS substrates. (**a**) SEM image of S-SiNRs@AuNPs substrate; (**b**) SEM image of T-SiNRs@AuNPs substrate; (**c**) SEM image of B-SiNRs@AuNPs substrate. All of the SiNRs have a diameter of ~200 nm and a length of ~0.8–1.2 μm for a single nanorod.

A scanning electron microscopy (SEM) image of uniform AuNPs with a diameter of ~20 nm is shown in Figure 2a. A strong absorption peak at 520 nm is observed from the solution of AuNPs measured by ultraviolet-visible (UV-Vis) absorption spectrum (Figure S1). Here, different methods were applied to conjugate the AuNPs at different positions of the SiNR. The diagrams of the three designed SiNRs@AuNPs substrates are shown in Figure 2b–d, respectively. The substrate of B-SiNRs@AuNPs was easily acquired by dropping AuNPs droplets (20 μL) onto the surface of the SiNRs for 24 h without the conglutination of MPTS. All of the AuNPs would sink to the bottom of substrate, as shown in the SEM image in Figure 3c. The methodology for making S-SiNRs@AuNPs substrates followed the steps described in the experimental section. Based on the SEM image in Figure 3b, abundant AuNPs have been successfully decorated on the side surface of SiNRs. The T-SiNRs@AuNPs substrate was obtained by high-temperature incubation and quick drying of the droplets. Interestingly, the AuNPs would stay on the top of SiNRs (Figure 3b) and form a layer of AuNPs film. All of the AuNPs located on a two-dimensional surface make a high-density AuNPs film with ultra-narrow gaps. Here, the period of the three types of SiNRs is 500 nm, which is large enough to form the gap-free field.

**Figure 4:**
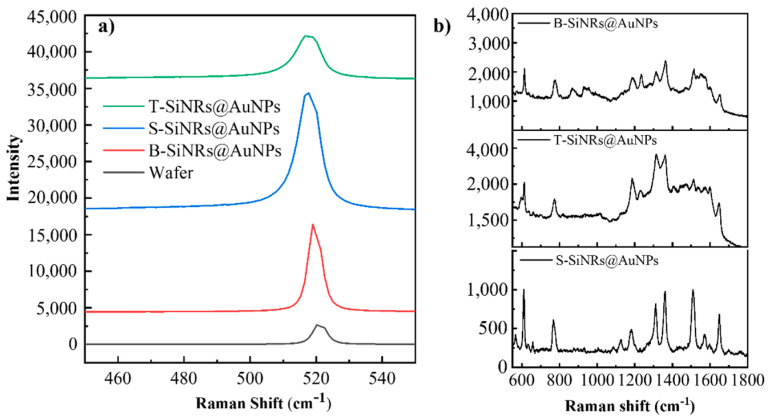
Raman measurements on different SERS substrates. (**a**) Si Raman signals (520.7 cm^−1^) from three types of substrates and Si wafer; (**b**) R6G Raman detection by B-SiNRs@AuNPs, T-SiNRs@AuNPs and S-SiNRs@AuNPs SERS substrates.

To investigate the sensitivity of the SiNRs@AuNPs substrate, firstly, enhancement of Si Raman signal by each SERS substrate was achieved. As shown in Figure 4a, the Si signals of the three types of substrates were compared under the same laser conditions (633 nm, ×10 objective, acquisition time 2 s), together with the planar Si wafer. Obviously, the SiNRs@AuNPs substrate obtained high enhancement at 520.7 cm^−1^. Specifically, the S-SiNRs@AuNPs substrate showed the strongest SERS sensitivity, reaching up to ~30 times compared with the plain wafer. We attribute this phenomenon to the intensive absorption of incident light by the ordered structures (enhanced reflection). After the conjugation of AuNPs, the Raman signal of Si was enhanced dramatically by the coupling of localized light and a strong localized surface plasmon resonance effects by AuNPs. Interestingly, the Raman signal of Si from T-SiNRs@AuNPs substrate was relatively weaker than the others. This is because the enhancement space is moved from the spatial space of the SiNRs to the surface of the SiNRs. As a result, the focus point of the laser was set on the surface of the SiNRs.

**Figure 5:**
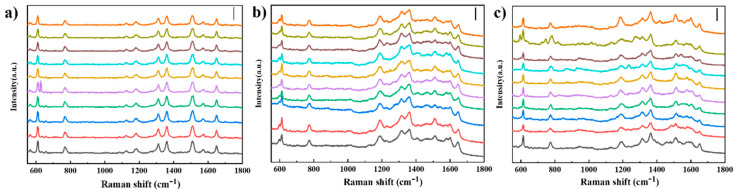
10 point Raman measurements on different SERS substrates. (**a**) R6G Raman detection by S-SiNRs@AuNPs, scale: 2000; (**b**) R6G Raman detection by T-SiNRs@AuNPs, scale: 4000; (**c**) R6G Raman detection by B-SiNRs@AuNPs, scale: 3000. The 10 measuring points were selected randomly to cover the sample.

Next, the designed substrates were applied to detect the signal of R6G molecule, a typical analyte for standard SERS testing. Due to the ultralow concentration of the R6G molecule, no sensitivity was found on the planar Si substrate. However, three types of SiNRs@AuNPs substrates acquired high SERS enhancement at a concentration of 10 ^−7^ M of the R6G molecule (Figure 4b). The typical Raman peaks of R6G (611 cm^−1^, 766 cm^−1^, 1312 cm^−1^ et al.) exhibited a large enhancement. Here, 10 Raman spectra were randomly obtained from each substrate and are shown in Figure 5. For the purposes of clear comparison, 10 Raman spectra are stacked from the bottom to the top of the chart, with a blank space of 20%. Unlike the results from the measurement of Si Raman signal (Figure 4a), the R6G Raman signal from S-SiNRs@AuNPs substrate exhibits the lowest intensity (also see in Figure 4b). To check the distribution of AuNPs of the S-SiNRs@AuNPs substrate, we find the density of the functionalized AuNPs on the side surface of SiNRs is the lowest. The SERS sensitivity could be increased dramatically with the reduce of the two-particle gap. With the adsorption of R6G molecules in the confined interstructure gaps, the Raman signal could be electromagnetically enhanced. Despite the low SERS sensitivity, significantly, the substrate achieved high reproducibility and spatial uniformity. In comparison, the Raman signal from the T-SiNRs@AuNPs substrate obtained the highest intensity, reaching 4 times higher than the S-SiNRs@AuNPs substrate. The 2D layer of AuNPs provides high density of hot spots. In addition, the different concentration of R6G molecules were measured by T-SiNRs@AuNPs substrate (Figure S2). The results show that the T-SiNRs@AuNPs substrate could detect molecules up to 10^−9^ M, which exhibit high SERS sensitivity. When the AuNPs are located on the bottom of the SiNRs, the substrate also achieves enhancement of the Raman signal, with the intensity being twice that of the S-SiNRs@AuNPs substrate.

**Figure 6:**
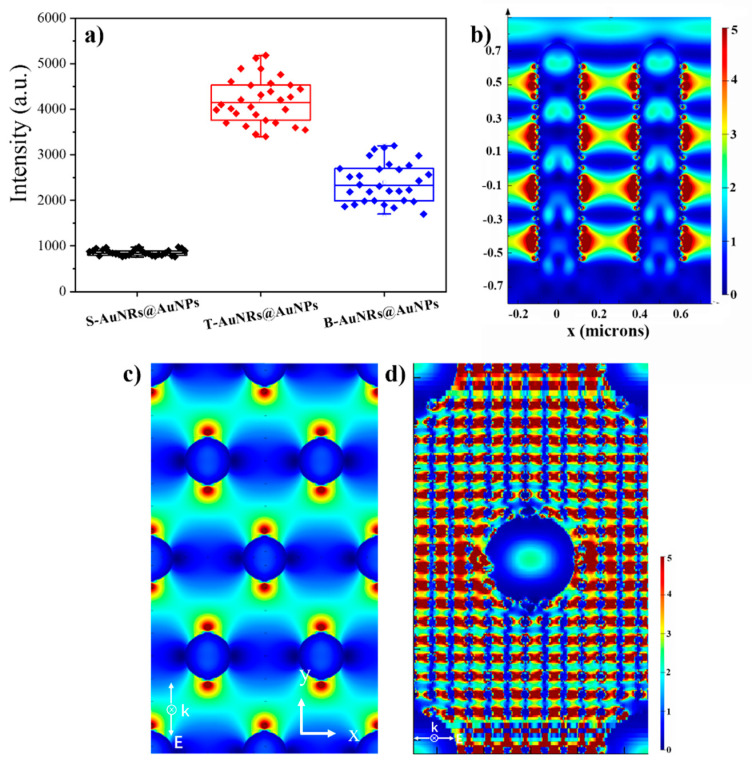
(**a**) Relative standard deviation of typical peak (1361 cm^−1^) from 10 Raman spectra; (**b**) FDTD simulation of the near-field electric field distribution of ordered SiNRs (left) and S-SiNRs@AuNPs (right) arrays. Both are side views of the structure. (**c**) Top view of the electric field distribution of S-SiNRs@AuNPs. (**d**) Top view of the electric field distribution of T-SiNRs@AuNPs.

To evaluate the SERS reproducibility of three types of SERS substrates, we calculated the relative standard deviation (RSD, the ratio of standard deviation to the mean) of noticeable peaks from three individual samples (each kind of substrate has 30 points), respectively. A typical peak analysis (1361 cm^−1^) is shown in Figure 6a. It can be seen that the RSD of S-SiNRs@AuNPs substrate reaches as low as 6.2%. The values of 30 points almost remain the same, although the points are measured randomly to cover a large area of the samples. The RSD of T-SiNRs@AuNPs and B-SiNRs@AuNPs substrate reached 16.2% and 13.4%, respectively. The typical enhancement factor (EF) of T-SiNRs@AuNPs substrate is 1.7 × 10^7^, reaching its strongest value in comparison with S-SiNRs@AuNPs and B-SiNRs@AuNPs substrates (calculated by the methods described in the SI). As a result, the ordered SiNRs with AuNPs on their side surface play an important role on the reproducibility of SERS signal while sacrificing high sensitivity, while the T-SiNRs@AuNPs substrate obtained the highest enhancement factor.

To elucidate the enhancement effects, we simulated the electric field distribution by means of finite-difference time-domain simulation (FDTD). The SiNR was set as ~1.3 μm in height, 200 nm in diameter and 500 nm in period (hexagonal-packed, model shown in Figure S3). The incident light is confined to different places with the change in wavelength [25,26]. We designed a structure that has an optimal spatial distribution, with an interstructure space below ~630 nm. The electric field distribution by SiNRs arrays and the S-SiNRs@AuNPs substrate was investigated. From the side view of SiNRs (Figure 6b), significantly, the interstructure space was dramatically enhanced by the spatially connected antinodes across the SiNRs array (in both SiNRs and S-SiNRs@AuNPs arrays). In addition, the areas around the AuNPs were further enhanced by LSPR, which were constructed by the coupling of AuNPs to AuNPs and ordered SiNRs@AuNP arrays. The decay of the electric field intensity at each antinode covers a distance of >125 nm, indicating the zero-gap field distribution between the ordered SiNRs horizontally. Corresponding to the distribution of SiNRs by SEM in Figure 3, the top view of the electric field intensity distribution is illustrated in Figure 6c by FDTD (incident light of polarization on the *y*-axis). It can be found that the antinodes are connected with their neighbors, which gives further evidence of the zero-gap electric field distribution. As a result, the great reproducibility of the S-SiNRs@AuNP SERS substrate would be attributed to the reasons of coupling of AuNP-induced LSPR and zero gap enhanced inter-rod space of the substrate by the light manipulation of ordered SiNR arrays. The same reason could be given for the T-SiNRs@AuNP SERS substrate (Figure 6d). The space will be greatly enhanced when the high density of 2D AuNPs layer is located in the right position (top of SiNRs arrays) of the localized antinode network. However, we found that the bottom antinode network could not be located on the surface ground of the SiNRs, even with a change in the height of the SiNR from 0.8 μm to 1.3 μm.

## 4. Conclusions

In summary, we fabricated hexagonal-packed SiNRs arrays and conjugated them with homogeneous AuNPs on the three typical positions of the SiNR. The SEM images clearly reveal the successful functionalization of AuNPs on the top, side and bottom of the SiNRs. With an optimized height, diameter, period, and location of AuNPs, three SiNRs@AuNPs SERS substrates exhibit stable and reproducible Raman signals of analyte molecules (R6G). Importantly, the S-SiNRs@AuNP substrate achieved high reproducibility and spatial uniformity (RSD as low as 6.3%). This is mainly contributed by the coupling of the sphere AuNPs, inducing a strong LSPR around the SiNRs, as well as the wide range of antinodes connected in the electric field between SiNRs. In addition, when the AuNPs were moved to the top of the SiNRs array in the 2D layer, the substrate achieves the lowest Si Raman signal, but the highest R6G SERS signal. The high density of AuNPs-AuNPs hot spots, coupled with the first layer of the antinode network, enhances the SERS detection dramatically. More importantly, the different SiNRs@AuNPs SERS substrates could be applied in the detection of different targets. For example, the S-SiNRs@AuNP substrate is suitable for macromolecules and proteins (such as DNA and fibrils), while the T-SiNRs@AuNP could be used in the ultra-high-sensitivity detection of chemical molecules (easily adsorbed on the AuNPs). Furthermore, the reported large-area SERS substrates can be easily fabricated by the low-cost methods of metal-assisted chemical etching and PS lithography. Overall, the ordered SiNRs@AuNPs array could serve as a highly sensitive and reproducible SERS substrate for label free molecule/biomolecule detection.

## Supplementary Materials

The following are available online at https://www.mdpi.com/2079-4991/10/7/1317/s1, Figure S1: Absorption of AuNPs measured by UV–Vis spectrum. Figure S2: Raman spectra of R6G molecules from 10^−6^ to 10^−9^ M. Figure S3: the FDTD model for simulation.
