# Supplementary material for: Spatial-Tunable Au Nanoparticle Functionalized Si Nanorods Arrays for Surface Enhanced Raman Spectroscopy
Source: Nanomaterials (Basel). 2020 Jul 4;10(7):1317. doi: 10.3390/nano10071317 (PMC7407171; doi:10.3390/nano10071317)
Supplement: Supplementary file 1 [file nanomaterials-10-01317-s001.pdf]

Supporting Information

Spatial-Tunable Au Nanoparticle Functionalized Si Nanorods arrays for Surface Enhanced Raman Spectroscopy

Dongdong Lin*, Kunjie Dai, Tianxiang Yu, Wenhui Zhao and Wenwu Xu*

Department of Microelectronic Science and Engineering, Department of physics, School of Physical Science and Technology, Ningbo University, 818 Fenghua Road, Ningbo 315211, China; lindongdong@nbu.edu.cn; 176000229@nbu.edu.cn; @nbu.edu.cn; 176001983@nbu.edu.cn; @nbu.edu.cn; zhaowenhui@nbu.edu.cn

* Correspondence: xuwenwu@nbu.edu.cn; lindongdong@nbu.edu.cn

1. Absorption of AuNPs measured by UV–vis spectrum

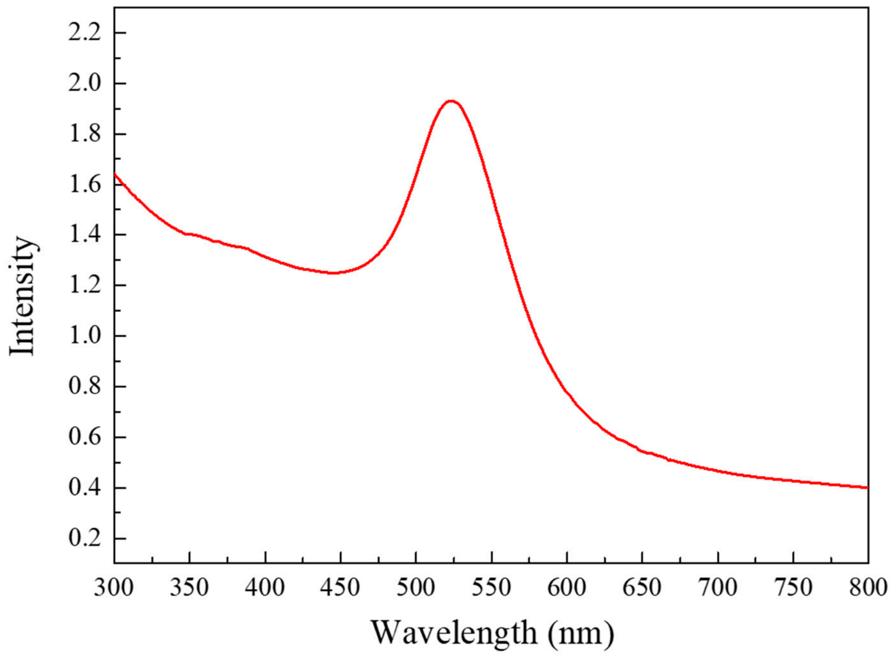

Figure S1. UV-Vis spectrum of fabricated AuNPs in water solution.

2. Raman spectra of R6G molecules from 10^{-6} to 10^{-9} M

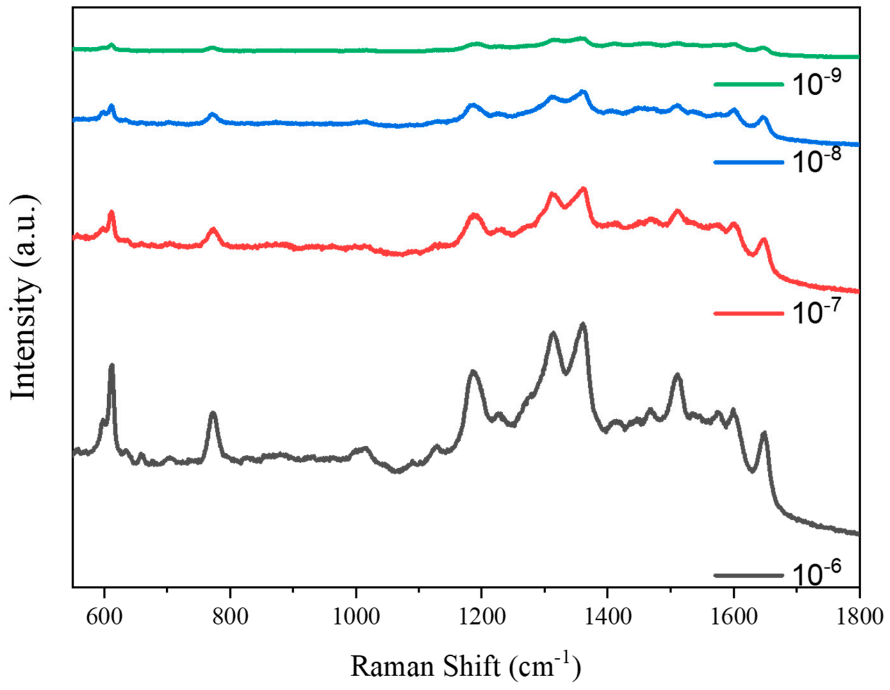

Figure S2. Raman spectra of R6G molecules from 10^{-6} to 10^{-9} M by T-SiNRs@AuNPs substrate.

3. The FDTD model for simulation

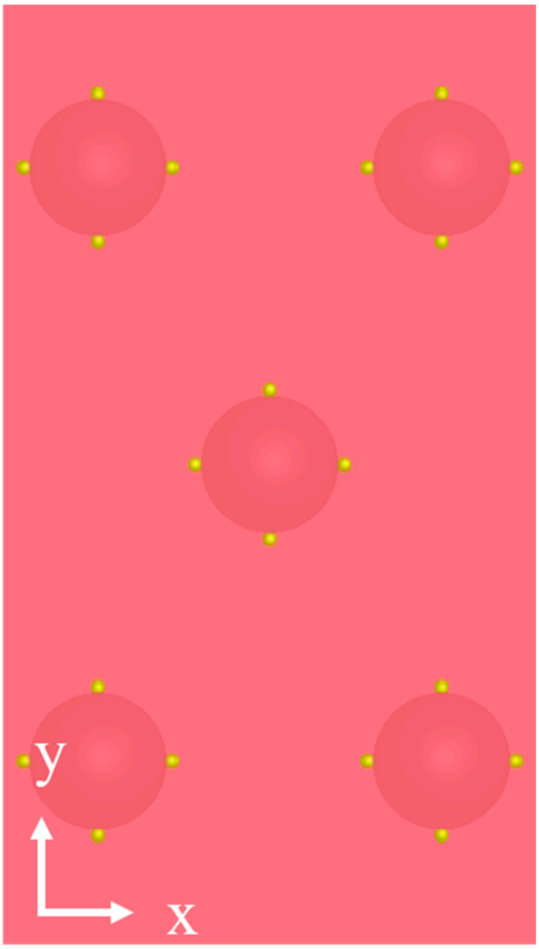

Figure S3. Top view of S-SiNRs@AuNPs array in FDTD simulation. Due to the calculation ability of computer, four directions along the z-axis of each SiNR were placed with AuNPs in 20 nm diameter.

4. The EF of R6G was calculated using the following formula with the enhanced Raman peak of 1361 cm^{-1}.

$$EF = \frac{I_{SERS}/(\mu_M \mu_S A_M)}{I_{RS}/(C_{RS} H_{eff})}$$

This formula is corrected for the solid periodic nanostructure SERS substrate with a rigorous definition of N_{Surf} *and* N_{Vol}.[1, 2]

I_{SERS} : the enhanced Raman intensity of measured molecules;

I_{RS} : the common Raman intensity of molecules without Raman enhancement.

μ_M : surface density of the individual SiNRs producing the enhancement and

μ_S : surface density of molecules on the metal.

A_M : surface area of one SiNR.

C_{RS} : the concentration of the used molecules in common Raman measurement.

H_{eff} : the effective height of scattering volume of the SiNRs.

In our case, the normal Raman spectrum was obtained from the probe volume of R6G solid film on clear silicon wafer.

Reference

1. Le Ru, E. C.; Blackie, E.; Meyer, M.; Etchegoin, P. G. Surface Enhanced Raman Scattering Enhancement Factors: A Comprehensive Study. *The Journal of Physical Chemistry C* **2007**, 111, 13794-13803.

2. Huang, J. A.; Zhao, Y. Q.; Zhang, X. J.; He, L. F.; Wong, T. L.; Chui, Y. S.; Zhang, W. J.; Lee, S. T. Ordered Ag/Si nanowires array: wide-range surface-enhanced Raman spectroscopy for reproducible biomolecule detection. *Nano Lett* **2013**, 13, 5039-45.
